# Obstetrical Nursing for Nurses and Students

**Published:** 1903-04

**Authors:** 


Obstetrical Nursing for Nurses and Students.—By Henry Enos Tuley, M. D., Louisville, Ky., Professor of Obstetrics, Kentucky University, Medical Department; Visiting Obstetrician to the John N. Norton Memorial Infirmary, Louisville City Hospital and the Home for Friendless Women, etc. Pages 202. Price, cloth, $1.00 net. G. P. Engelhard & Company, Chicago, 1902.

